# Phenotypical Conversions of Dermal Adipocytes as Pathophysiological Steps in Inflammatory Cutaneous Disorders

**DOI:** 10.3390/ijms23073828

**Published:** 2022-03-30

**Authors:** Ilja L. Kruglikov, Zhuzhen Zhang, Philipp E. Scherer

**Affiliations:** 1 Scientific Department, Wellcomet GmbH, 76646 Bruchsal, Germany; i.kruglikov@wellcomet.de; 2 Touchstone Diabetes Center, Department of Internal Medicine, University of Texas Southwestern Medical Center, Dallas, TX 75390-8549, USA; zhuzhen.zhang@utsouthwestern.edu

**Keywords:** dermal adipocytes, de-differentiation, re-differentiation, trans-differentiation, psoriasis, acne

## Abstract

Adipocytes from the superficial layer of subcutaneous adipose tissue undergo cyclic de- and re-differentiation, which can significantly influence the development of skin inflammation under different cutaneous conditions. This inflammation can be connected with local loading of the reticular dermis with lipids released due to de-differentiation of adipocytes during the catagen phase of the hair follicle cycle. Alternatively, the inflammation parallels a widespread release of cathelicidin, which typically takes place in the anagen phase (especially in the presence of pathogens). Additionally, trans-differentiation of dermal adipocytes into myofibroblasts, which can occur under some pathological conditions, can be responsible for the development of collateral scarring in acne. Here, we provide an overview of such cellular conversions in the skin and discuss their possible involvement in the pathophysiology of inflammatory skin conditions, such as acne and psoriasis.

## 1. Introduction

Dermal white adipose tissue (dWAT) is a newly recognized fat depot located in the superficial hypodermis [1,2]. Recent findings have revealed that dermal adipocytes are phenotypically different from their anatomically nearby subcutaneous counterparts and demonstrate diverse rapid transformations in response to some external and internal factors. These cells contribute in substantial ways to a variety of processes. This includes cutaneous events, such as hair follicle (HF) cycling, wound healing, and the innate immune response (mainly through the production of antimicrobial peptides); additionally, we suggested that dermal adipocytes are involved in scarring and skin aging [1]. One unique property of murine dWAT is the remarkable oscillation of its volume during the hair follicle cycle; it strongly decreases in catagen and completely restores in anagen [2,3,4]. The physiological impact and underlying reasons for these oscillations have been a topic of intensive discussion in the recent past.

**Figure 1:**
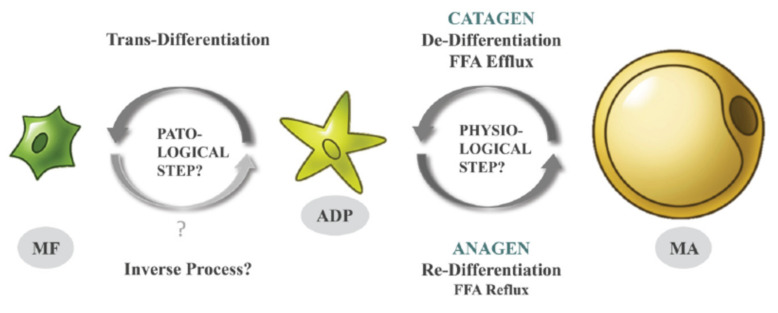
De-, re-, and trans-differentiation of dermal adipocytes during the HF cycle [9] (with permission from Experimental Dermatology).

Recently, we demonstrated, in murine models, that these oscillations are the result of specific cellular conversions; mature dermal adipocytes de-differentiate in the catagen phase into the much smaller fibroblast-like adipocyte-derived preadipocytes (ADPs) and re-differentiate in anagen back into mature adipocytes (Figure 1) [3,4]. Such physiological processes of de- and re-differentiation of adipocytes repeat during every HF cycle and involve a large number of murine dermal adipocytes. The remaining mature adipocytes in the dWAT layer may be recruited from distinct adipose progenitors that were not subject to such previous cyclic conversions.

De-differentiation of dermal adipocytes into ADPs in the catagen is accompanied by a significant reduction of their volumes and leads to loading of the dWAT and reticular dermis with fatty acids [5,6]. Re-differentiation of preadipocytes in anagen leads to a regain of the cell volumes and causes intensive expression of the anti-microbial peptide cathelicidin. This expression can be significantly enhanced in the presence of pathogens [7]. Local loading of the skin with extracellular lipids and cathelicidin can induce a local inflammatory reaction in the affected skin areas.

Moreover, under appropriate conditions, ADPs can leave this cycle and transdifferentiate into synthetically highly active myofibroblasts. While the specific signals triggering such trans-differentiation still remain to be elucidated, they do involve stimulation by transforming growth factor beta (TGF-β). This type of cellular change does not occur under healthy conditions and can lead to local cutaneous fibrosis and scarring (Figure 1). Whereas some authors found that myofibroblasts may re-differentiate into adipose cells [8], this issue will need further investigation.

Whereas the cell conversions described in [4] directly affect only the local structure and physiology of dWAT, their effects can be transmitted to the dermis and even the epidermis and can thus be involved in the pathophysiology of different cutaneous conditions. Here, we address the question of how these cellular changes can influence the pathophysiology of common inflammatory skin disorders, such as acne and psoriasis.

## 2. DWAT Layer in Human Skin

dWAT has been extensively investigated and well described in murine skin, whereas the existence of this layer in humans is a matter of controversy [1]. The main problem is the obvious inability to conduct lineage-tracing studies in humans [10]. Another problem is the widespread opinion that there is no anatomical demarcation of dWAT in human skin analogous to the *Panniculus carnosus* (a layer of striated muscle cells) seen in rodents. Some authors have proposed that this structure is underdeveloped in humans [2]. In contrast, others describe fascial planes composed of fibrous connective tissue and muscular fibers producing a separating layer between the dermis and hypodermis, which can be considered a structure homologous to the *Panniculus carnosus*. Confusingly, these layers have different designations depending on anatomical location, such as *Galea aponeurotica* in the head, *superficial musculo-aponeurotic system* (SMAS) in the face, *platysma* in the neck, *Scarpus fascia* in the abdomen, and *Colles fascia* in the perineum [11].

Nevertheless, several authors have proposed the existence of a similar dermal-associated fat depot in humans and described its unique geometry as a flat superficial layer of subcutis with additional adipose cones penetrating the dermis and located around hair follicles (HFs) [1,2,12]. At the same time, this does not mean that dWAT in rodents and humans is anatomically, physiologically, and metabolically identical [4]. To avoid possible confusion, some authors have proposed to designate dWAT in humans as skin-associated adipose tissue (SAAT) [4] or skin-associated fat (SAF) [13].

A possibility is to use chemical shift magnetic resonance tomography to separate the fat-only and water-only images and thus to assess the dWAT depot in rodents and the SAF depot in humans [13,14]. Applying this method, these authors estimated the thickness of the SAF as a body-wide depot to be about 10.05 ± 2.45 mm (females) and 6.27 ± 1.89 mm (males) with a high inter-individual variability of thicknesses. A similar dimorphism was earlier observed in murine dWAT [15] and later confirmed in [13]. No significant correlation was found between the thickness of this layer and the age of the individuals. In humans, the thickness of SAF is independent of the body mass index (BMI) in males and nearly independent of this parameter in females [13], whereas in murine models, dWAT thickness positively correlates with BMI [4]. Interestingly, while adipocytes from dWAT are resistant to β-adrenergic-induced lipolysis [4,13], adipocytes from SAF were found to be highly lipolytic [13].

Combined, there is overwhelming evidence that humans have a skin-associated adipose tissue depot similar to dWAT in rodents. Given that adipocytes from dWAT are involved in various important physiological and pathological processes in the skin, we assume that the same is correct for skin-associated adipocytes in humans.

## 3. Connection of Acne and Psoriasis to the HF Cycle and High Fat Diet

### 3.1. Acne vulgaris

*Acne vulgaris* is the most common inflammatory skin disorder with a high prevalence in adolescence, and it is spatially localized to the small skin areas adjacent to pilosebaceous units. The pathophysiology of acne is connected to etiopathogenic factors, such as hyperseborrhea, follicular hyperkeratinization, and the pathogenic behavior of the gram-positive bacterium *C. acnes* [16]. Hyperseborrhea was traditionally linked to increased sebum excretion, whereas follicular hyperkeratinization was explained by the abnormal differentiation and desquamation of keratinocytes. The pathogenic behavior of *C. acnes* was mainly explained by the uncontrolled proliferation of these bacteria [9]. Remarkably, perifollicular inflammation typical of acne is found just in the earliest stages of the development of acne lesions [17], which means that *Acne vulgaris* should be considered a primary inflammatory skin disorder.

The relative contribution of exposure to high-fat diets (HFDs) toward acne is still a matter of controversy. In fact, a recent report argued that adiposity in adolescence is inversely related to acne [18]. At the same time, the mammalian target of rapamycin complex 1 (mTORC1) expression, known to be regulated by HFD, growth factors, and stress and critically involved in the induction of lipolysis in adipocytes, is significantly increased in acne lesions compared to non-lesional skin [19]. HFD also efficiently induces the expansion of dWAT [4]. This suggests that HFD can theoretically influence acne not through a systemic impact but rather through the local modification of the adjacent dWAT. However, other factors cannot be excluded.

*Acne vulgaris* is not only spatially connected to pilosebaceous units, but it is also strongly dependent on the HF cycle. The initiation of acne lesions was historically assigned to the catagen/telogen phases of the HF cycle [20]. At the same time, there is no known HF cycle-dependent change in *C. acnes* behavior. Moreover, whereas the sebaceous gland indeed demonstrates varied sebum production in different HF phases, this secretion is normally lower in catagen than in anagen, which argues against predominant production of acne lesions during catagen/telogen phases. This paradox is difficult to explain in the frames of the classical acne pathophysiology, primarily connecting perifollicular inflammation in acne lesions with hyperseborrhea and activity of *C. acnes*. On the other hand, we can explain the phenomenon through temporarily restricted loading of perifollicular areas with free fatty acids released during de-differentiation of mature adipocytes into ADPs in the catagen phase of the HF cycle (Figure 1). Moreover, trans-differentiation of ADPs into myofibroblasts can explain the appearance of hypotrophic scars in long-lasting acne lesions.

### 3.2. Psoriasis vulgaris

Psoriasis is a common chronic autoimmune hyperproliferative skin disorder with a strong genetic predisposition. Whereas the pathophysiology of psoriasis is not fully elucidated, it is widely accepted that it includes a dysregulation of innate and adaptive skin immunity, epidermal hyperproliferation, and inflammation. The clinically relevant skin modifications in psoriasis were initially found in the epidermis and connected to the pathological behavior of keratinocytes. For this reason, psoriasis has long been considered an inflammatory and hyperproliferative disease of the epidermis.

Psoriasis is considered to be independent of HF cycling. However, remarkable correlations between epidermal proliferation in psoriatic lesions and the anagen phase of the HF cycle were first discussed in [21]. These authors proposed that processes underlying both HF cycling and psoriasis share the same “switch-on” mechanisms. HFs within psoriatic scales demonstrate a remarkable shift in the relative distribution of HFs in the different phases; there are almost twice as many telogen HFs in psoriatic skin [22].

Such a modification in the staging of HF distribution points to a partial synchronization of HFs in psoriatic lesions. This is surprising, since human HFs under normal physiological conditions demonstrate highly asynchronous behavior. However, as we argued previously, HF synchronization can take place in some spatially restricted areas [23], which can also lead to synchronized changes in dermal adipocytes. 

Obesity demonstrates multiple comorbidities, and psoriasis is one of them [24]. Epidemiological studies highlight that obesity and weight gain are important risk factors for psoriasis [25,26]. On the other hand, weight loss has a positive impact on the severity of the disease [27]. Specifically, abdominal fat is intricately associated with disease predisposition and progression, and the metabolic health of visceral adipose tissue strongly relates to the manifestations of psoriasis. Skin and adipose tissue interact through adipokines. One of these adipokines—adiponectin—is a well-known regulator of insulin sensitivity, which also modulates proliferation and migration of keratinocytes [28] and modifies psoriasiform skin inflammation through suppression of IL-17 production [29]. Consistent with these observations, adiponectin KO mice can demonstrate a severe form of psoriasiform dermatitis [29]. On the other hand, HFD significantly exacerbates early psoriatic skin inflammation independent of obesity. Furthermore, a reduction of dietary saturated fatty acids improves psoriasiform inflammation independently of weight and fat content [30].

We have proposed that it is not the abdominal adipose tissue; rather, it is the dWAT located at the interface between dermis/subcutis that is involved in the pathophysiology of psoriasis [31,32]. There are several direct indications that dWAT undergoes structural modifications strongly associated with the psoriatic phenotype. PUVA treatment (a combination treatment consisting of application of psoralens (light sensitizers) (P) and then exposing the skin to UVA (long wave ultraviolet radiation) of K5.hTGFb1 transgenic mice (a murine model for psoriasis) caused a significant reduction of the dWAT layer beneath the psoriatic lesions, and such a modification correlated with clinical skin improvements in these animals [33]. Sonoelastographic investigation of the fat tissue located beneath the human psoriatic plaques demonstrates that the mechanical properties of the subcutis, beneath these spatially restricted areas, are very different from those in the areas covered by healthy skin. Successful treatment of psoriasis leads to skin improvements and simultaneous normalization of the adjacent fat tissue [34,35].

**Figure 2:**
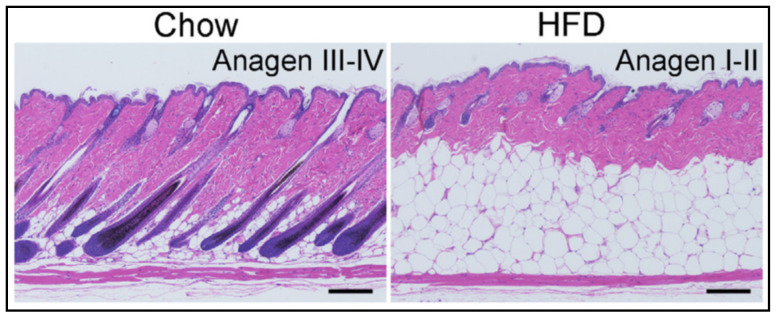
Influence of the high-fat diet on the dWAT structure and HF cycle [4] (with permission of The Journal of Clinical Investigation).

Taking into account the spatially limited distribution of psoriatic lesions and the modifications of dWAT located beneath these lesions [31,32], a critical question is what happens with dWAT during exposure to HFD? As we have demonstrated recently, HFD prompts at least two major modifications to dWAT: (1) a dramatic expansion of its volume involving both the processes of hyperplasia and hypertrophy of dermal adipocytes, and (2) a partial synchronization of HFs in the catagen/telogen phases with an impairment of their re-entry into anagen compared to chow-fed controls [4] (Figure 2). Both modifications effectively increase the number of spatially and temporally correlated de-differentiation and re-differentiation events that mature adipocytes undergo. Consequently, the relative levels of lipids in skin in the catagen stage of the HF cycle, as well as the production of the antibacterial cathelicidin protein in the anagen, differ. Of note, such dWAT modifications also modulate the mechanical properties of the skin.

Many of these pathological changes discussed here may be relevant to other skin diseases beyond Acne and Psoriasis. This includes *hidradenitis suppurativa* and other pathological alterations in the skin. Future experimental approaches will have to put the underlying mechanistic connections that we propose here into the test.

## 4. Reduction of Lipid Droplets in Mature Adipocytes during Their De-Differentiation into ADPs

Elevation of the free fatty acid (FFA) concentration induces oxidative stress and has a proinflammatory effect [36]. Correspondingly, loading of reticular dermis with lipids in the catagen phase of the HF cycle observed in [4,5] has to lead to local inflammation; the physiological process of de-differentiation of dermal adipocytes can provide a pathological microenvironment at the distal end of the HF, making the skin inherently more susceptible to inflammation. At the same time, the FFAs secreted from the dedifferentiating cells into the extracellular space and thus the associated inflammation are critically dependent on the reduction of lipid droplets.

Lipid droplets, which for a long time have been considered inert storing repositories for neutral lipids, are now recognized as highly dynamic organelles significantly contributing to lipid metabolism by buffering potentially toxic lipids [37]. They may also be involved in inflammatory responses through the release of eicosanoids. Some important pro-inflammatory lipid mediators, such as the non-classical eicosanoids hepoxilins (derivatives of arachidonic acid), as well as related lipids, are indeed upregulated in psoriatic lesions [38,39,40]. For example, hepoxilin B3 was found to be increased 16-fold in psoriatic scales compared to the normal epidermis [38]. Of note, inhibition of the eicosanoid leukotriene B4 provided a significant reduction of inflammation in acne, supporting the idea that lipids are direct inducers of inflammation in acne, independently of *C. acnes* [41]. At the same time, arachidonic acid is implicated in adipogenesis, modulating the differentiation potential of preadipocytes [42]. Consequently, lipid microdroplets are extruded into the extracellular space and can induce an inflammatory response in the surrounding tissue.

The reduction in lipid droplet size is the most prominent modification of the overall cell structure during de-differentiation of mature adipocytes into ADPs [4]. Lipid droplets are composed of a hydrophobic core consisting mainly of triacylglycerols and cholesteryl esters, surrounded by a stabilizing phospholipid monolayer containing embedded proteins, among them perilipins (PLIN). Two types of perilipins, PLIN1 and PLIN2, are known to be responsible for the stability of lipid droplets. PLIN1 remains undetectable in preadipocytes, but its content remarkably increases during adipocyte differentiation; PLIN2 content is highest in preadipocytes and is strongly downregulated during differentiation of preadipocytes into mature adipocytes and is almost undetectable in mature cells [43].

**Figure 3:**
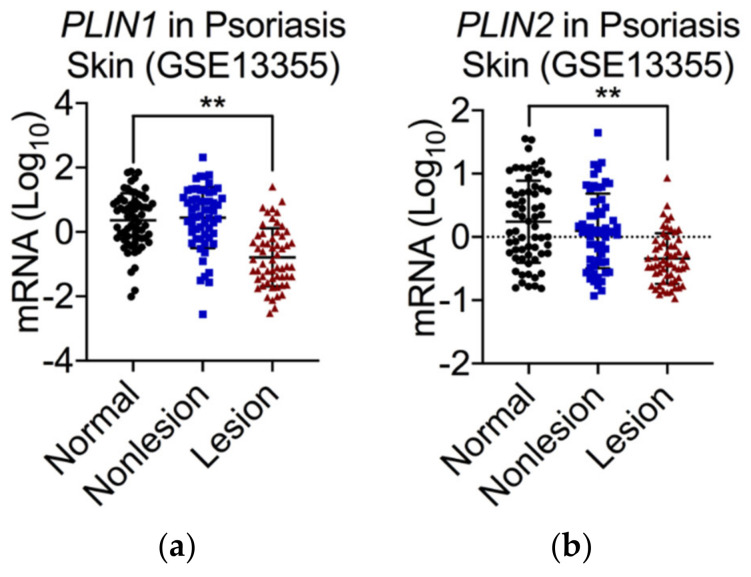
mRNA expression for PLIN1 (**a**) and PLIN2 (**b**) in psoriatic scales compared to normal and non-lesional skin. The materials and methods were described in [4], ** *p* < 0.01.

Both PLIN1 and PLIN2 are significantly reduced in psoriatic lesions compared to normal and non-lesional skin [4] (Figure 3). Additionally, adiponectin, which is an anti-inflammatory adipokine, is strongly downregulated in psoriatic lesions compared to normal or non-lesional skin, whereas the production of cathelicidin in these lesions is significantly increased [4]. The likely explanation for these transcriptional changes may be the accumulation of cells that spend an extended period of time in the process of differentiation, i.e., have left the pre-adipocyte stage behind but have not yet reached the fully differentiated state. In other words, they have an extended re-differentiation time. This phenomenon can explain the following changes seen in psoriatic scales:

1. Reduction of both PLIN1 (highly expressed only in fully mature adipocytes with big lipid droplets) and PLIN2 (highly expressed only in preadipocytes);
2. Reduction of adiponectin and leptin (expressed only in mature adipocytes);
3. Overproduction of cathelicidin (occurs during differentiation of preadipocytes into mature cells and is significantly enhanced in the presence of cutaneous pathogens).

The main pathways that catabolize lipid droplets are lipolysis and lipophagy, both of which are activated during prolonged nutrient deprivation [44]. Lipolysis releases fatty acids and glycerol from lipid droplets through the action of lipases. Lipophagy prompts proteins and organelles to be delivered from autophagosomes to lysosomes for degradation, and the products of degradation are normally released into the cytoplasm for reuse [45]. Whereas lipolysis and lipophagy share some similarities (they are both catabolic pathways activated in response to nutrient deprivation), they are not directly interrelated [46]. Lipophagy depends on cellular lipid content and significantly decreases with increased cellular lipid content. Additionally, the activation of lipophagy during starvation initially leads to an increase in the number and volume of lipid droplets in adipocytes to prevent lipotoxic damage to mitochondria [47]. Remarkably, inhibition of autophagy in 3T3-L1 preadipocytes by knocking down ATG5 or ATG7 blocked the differentiation of these cells into mature adipocytes [48]. Of note, 3T3-L1 cells demonstrate a striking similarity to dermal adipocytes and have a high level of cathelicidin expression upon differentiation [7].

Lipophagy may be responsible for a reduction of dWAT in the catagen phase of the HF cycle [49]. These authors report that mature dermal adipocytes adjacent to HFs contain lipid droplets with large circular vacuoles, similar to those observed in yeast during autophagy. Furthermore, they display a significantly increased content of microtubule-associated proteins 1A/1B light chain 3B (LC3B), which is typically present in autophagosome membranes [49]. However, psoriasis is characterized by impaired autophagy; LC3 expression is strongly reduced in non-lesional psoriatic skin and undetectable in psoriatic scales [50], which speaks against the role of autophagy in psoriasis.

Both lipolysis and lipophagy can lead to a reduction in lipid droplets through intracellular degradation. Two alternative mechanisms related to the release of intact portions of lipid droplets from cells were proposed. These mechanisms can shift the degradation of lipid droplets from the intracellular to the extracellular space and thus additionally promote local tissue inflammation. The first of these mechanisms is the secretion and transport of exosomes loaded with lipids or small fragments of lipid droplets. Extracellular lipids can locally regulate adipose tissue macrophages. However, the lipid catabolism in the lysosomes of these macrophages is independent of autophagy, which suggests that catabolized lipids do not come from the lipid droplets located within macrophages [51]. A reasonable explanation for this finding is that adjacent mature adipocytes serve as the source of lipids for tissue macrophages. Indeed, adipocytes can release small exosomes containing lipid droplets, as well as associated proteins, such as adipose triglyceride lipase and PLIN1, and under normal physiological conditions, this release can be as high as 1–2% of the total lipid content per day [52]. Another mechanism for a quick reduction of lipid droplets in mature adipocytes during their de-differentiation may be the fragmentation of the lipid droplet and the exocrine secretion of these fragments [53,54]. De-differentiation of mature adipocytes both in vitro and in vivo occurs through an intermediate step production of adipocytes with multilocular lipid droplets from unilocular cells [55]. De-differentiation of mature porcine and human adipocytes in a ceiling culture to fibroblast-like cells was reported to be accompanied by release of the lipid droplet fragments [53,54,56]. In human abdominal adipose tissue samples obtained from obese patients, less than 1% of adipocytes demonstrated such a release [54]. However, this rate may be significantly higher in the physiologically much more active dWAT depot. Morphologically, this mechanism is similar to the apocrine secretion described in alveolar cells of the mammary gland [54], where the de- and re-differentiation of adipocytes during and after lactation was formally demonstrated in vivo [57]. Remarkably, PLIN1 and PLIN2 appear to be involved in the regulation of this secretory step [58], which allows for a quick and comprehensive loss of lipids from mature adipocytes and leads to the appearance of isolated lipid droplets in the extracellular space, able to promote tissue inflammation. Future research will be needed to demonstrate the possible involvement of this mechanism in psoriasis.

## 5. TGF-β1 and Cav-1 Expressions in Acne and Psoriasis

Transforming growth factor beta (TGF-β) is a well-known master regulator of collagen production. TGF-β receptors co-localize in the plasma membrane with caveolin-1 (Cav-1) in the presence of high molecular weight hyaluronan [59]. However, high levels of TGF-β can induce cutaneous inflammation and hyperproliferation of keratinocytes [60]. This is a state that corresponds to low-level expression of Cav-1 in different hyperproliferative and inflammatory skin conditions. This includes psoriasis, which we discussed in detail recently [9,61,62,63].

TGF-β1 is potently increased in homogenized acne lesions [64]. TGF-β1 is also increased in both the serum and skin of psoriasis patients [65]. In addition, there is a correlation between TGF-β1 levels and the development of inflammatory reactions/psoriatic phenotypes [66,67].

Since TGF-β1 is an important factor involved in the de-differentiation of mature adipocytes, such correlations between TGF-β1 and Cav-1 should point to the involvement of Cav-1 in the processes of de-, re-, and trans-differentiations of dermal adipocytes. Indeed, it is well established that a reduction of Cav-1 expression is typical during de-differentiation of mature adipocytes (which generally have much higher Cav-1 expression than their dedifferentiated counterparts), as well as in trans-differentiation of ADPs into myofibroblasts, which are characterized by a very low Cav-1 level, consistent with their high synthetic activity for collagen production. Of note, low Cav-1 expression is also characteristic in adipocytes with active lipolysis [68].

## 6. How Are the Cellular Transformations of dWAT Involved in the Pathogenesis of Acne?

De-differentiation of dermal adipocytes in the catagen phase of the HF cycle provides a significant degree of lipid exposure in the areas adjacent to the distal parts of the HFs [4]. Thus, it can trigger a rapid local inflammatory response. Moreover, the temporally restricted appearance of de-differentiated adipocytes in the catagen phase can significantly change the local interactions of *C. acnes* with host cells. Indeed, for fibronectin-binding bacteria, host–pathogen interactions are dependent on the Cav-1 content of host cells [9,69].

The involvement of de-differentiating dermal adipocytes in ADPs explains why acne lesions are found to be associated with the catagen/telogen phases of the HF cycle [20]. Progressive chronological aging is associated with a reduced prevalence of acne. Whereas adolescent individuals have the highest prevalence of *Acne vulgaris*, exceeding 85%, this prevalence was found to be 51%/43% for females/males in the age group of 20–29-year-olds, 35%/20% for 30–39-year-olds, 26%/12% in 40–49-year-olds, and 15%/7% in the group of individuals older than 50 years of age [70]. The reduction in acne prevalence with chronological aging was traditionally explained by reduced sebum production. On the other hand, the number of cellular de-differentiation events in dWAT also progressively reduces with aging [71], which leads to a corresponding reduction of excreted lipids during the catagen phase.

We also appreciate that prolonged inflammation in acne often leads to the development of acne scars, more than 70% of which are atrophic. Such acne scarring is mainly connected to a local reduction in the elastic collagen networks in the affected skin. However, recently it was reported that TGF-β1 expression, which is generally high in initial acne lesions, is normalized after 3 days in the sub-group without scarring but remains high even after 7 days in the sub-group with scarring [72]. Such prolonged activation of TGF-β1 in acne lesions can lead to enhanced trans-differentiation of preadipocytes into myofibroblasts, with consequent production of fibrosis and scarring. Indeed, H&E staining of the biopsies from normal human skin and atrophic scars reveals a complete disappearance of dWAT in the scarring area [73], which additionally supports the involvement of trans-differentiation of dermal adipocytes in the formation of atrophic scars. However, we should note that fibrosis does not always occur in the post-inflammatory state. For example, in the course of HFD, fibrotic streaks in the adipose tissue compartment appear prior to the onset of inflammation [74].

## 7. How Are Cellular Dedifferentiation Events in dWAT Involved in the Pathogenesis of Psoriasis?

De-differentiation of dermal adipocytes is connected with a loading of the dermis with lipids in the catagen phase of the HF cycle. A high level production of cathelicidin characterizes the early stages of adipogenesis, associated with the re-differentiation of preadipocytes into mature adipocytes in the anagen phase [4]. The strong loading of the skin with secreted lipids at the distal end of the HFs can lead to the induction of spatially and temporally limited inflammation in this area. Cathelicidin also demonstrates pro-inflammatory aspects and is dramatically increased in human psoriatic lesions [4]. This, together with further synchronization of the HF cycle, can lead to a spatially correlated inflammatory response in the skin, spreading the inflammatory effect from an individual HF to the microenvironment in the interfollicular space.

Generally, this proposed mechanism can reflect the widespread prevalence of ADPs in psoriatic skin. This assumption is consistent with the very low level of Cav-1 reported in psoriatic lesions [61]. Of note, the de-differentiated adipocytes have the potential to induce the formation of a basement membrane with typical *rete ridge* structures during wound healing [75]. Taking into account that *rete ridges* are strongly enhanced in psoriatic skin, one is tempted to speculate that this phenomenon is closely connected to the increased ratio of ADP to mature adipocytes in psoriatic scales.

Epidermal cells significantly elevate their mitochondrial uptake of fatty acids and enhance β-oxidation in an infection-induced model of cutaneous inflammation, contributing to the recruitment of leucocytes [76]. Release of fatty acids by de-differentiating adipocytes significantly modifies the metabolic substrates processed by keratinocytes and fibroblasts, thus providing a strong impetus for their metabolic reprogramming [5,6]. Such reprogramming significantly influences the synthetic and proliferative capacities of these cells. Indeed, carnitine palmitoyltransferase-1 (CPT-1)—A key enzyme for the transport of long-chain fatty acids into mitochondria—is overexpressed in psoriatic lesions. This reflects the increased demand for fatty acid oxidation in lesional skin [77]. Moreover, inhibition of CPT-1 provided a significant reduction in keratinocyte proliferation and differentiation, demonstrating an anti-psoriatic effect [77].

Possible inflammatory skin reactions caused by a local secretion of lipids/cathelicidin in the catagen/anagen phase of the HF cycle, concomitant with the de- and re-differentiation of dermal adipocytes, raise the question as to which phase of the HF cycle is mainly connected with the initiation of psoriatic scales? This important question remains to be answered in future research. At the same time, no association with scarring/fibrosis has been found in psoriasis. This clearly indicates that the de-differentiated adipocytes do not morph into myofibroblasts in psoriatic lesions, in contrast to what is observed under other conditions, such as sclerosis. The reason for this will also need to be elucidated in future research.

## 8. Can Acne and Psoriasis Be Considered Two Sides of the Same Coin?

Whereas both acne and psoriasis have a range of associations with other diseases, no direct relationship between these two skin conditions has been found to date. This is surprising in light of the fact that the same pathophysiological mechanisms connected with cell de-differentiation in dWAT are expected to be present in both skin conditions. There are additional apparent contradictions with respect to the opposite prevalence of acne and psoriasis in different ethnic groups. In fact, psoriasis demonstrates an almost 50% lower prevalence in African Americans compared to Caucasians [78], whereas the prevalence of acne in African American females was reported to be about 50% higher than in Caucasian Americans [79].

This apparent contradiction is related to the fact that African Americans have significantly lower hair density and growth rates but a higher frequency of terminal HFs in the catagen/telogen phases than Caucasians [80]. Such a shift in the distribution of HF phases leads to different levels of lipid loading during de-differentiation events of dermal adipocytes in the catagen phase and, correspondingly, modifies the production of cathelicidin during re-differentiation of ADPs into mature adipocytes in the anagen phase. As discussed above, the first process is involved in the pathogenesis of acne, leading to an established lower prevalence in African Americans. In contrast, the second process is of importance in psoriasis, leading to the lower prevalence of this disease in the same ethnic group.

Indeed, it has been reported that African Americans produce significantly less cathelicidin (63% less in experiments in vitro) than Caucasians [81]. This effect was so far connected to a high level of melanin and correspondingly low levels of vitamin D (usually activating the cathelicidin production in the skin) in the skin of this ethnic group. However, dWAT significantly expands in the absence of vitamin D [82], which is connected to the enhanced differentiation of murine preadipocytes [9,83].

Additionally, African Americans have elevated levels of TGF-β1 expression in the skin [84], which corresponds to the observed lower levels of Cav-1, correlating with a high prevalence of hypertrophic scars, keloids, and systemic sclerosis in African Americans compared to Caucasians [62]. Such modified expression of TGF-β1 and Cav-1 may, among others, point to a modified ratio of immature-to-mature adipocytes in the affected dWAT layer.

From this point of view, acne and psoriasis appear as two sides of the same coin, and it should be possible to modulate them by similar treatment interventions, influencing the cell conversion events in dWAT. This important postulate will need to be carefully tested experimentally in future research.

## 9. Conclusions

Cyclic de- and re-differentiation of dermal adipocytes can significantly influence the development of skin inflammation under different cutaneous conditions, including acne and psoriasis. This inflammation can be connected to local loading of the reticular dermis with lipids released in the catagen phase of the HF cycle (as in acne) or with a widespread release of cathelicidin, typically taking place in the anagen phase (especially in the presence of pathogens), such as in psoriasis. Additionally, trans-differentiation of dermal adipocytes into myofibroblasts, which can occur under some pathological conditions, can be responsible for the development of collateral scarring in acne. Long-lasting exposure to a high fat diet significantly modulates the dWAT volume, increasing the number of synchronously differentiating cells and thereby promoting a high level of local inflammation in the skin.

## Data Availability

Not applicable.
